# Supplementary material for: Impaired Response to Mismatch Novelty in the Li2+-Pilocarpine Rat Model of TLE: Correlation with Hippocampal Monoaminergic Inputs
Source: Biomedicines. 2024 Mar 12;12(3):631. doi: 10.3390/biomedicines12030631 (PMC10968540; doi:10.3390/biomedicines12030631)
Supplement: Supplementary file 1 [file biomedicines-12-00631-s001.zip › biomedicines-2887930-supplementary.pdf]

Impaired Response to Mismatch Novelty in the Li^{2+}-Pilocarpine Rat Model of TLE: Correlation with Hippocampal Monoaminergic Inputs.

Carlos Nascimento, Vasco Guerreiro-Pinto, Seweryn Pawlak, Ana Caulino-Rocha, Laia Amat-Garcia and Diana Cunha-Reis *

*Correspondence: Departamento de Biologia Vegetal, Faculdade de Ciências, Universidade de Lisboa, Campo Grande, 1749-016 Lisboa, Portugal; dcreis@ciencias.ulisboa.pt

Supplementary Figures:

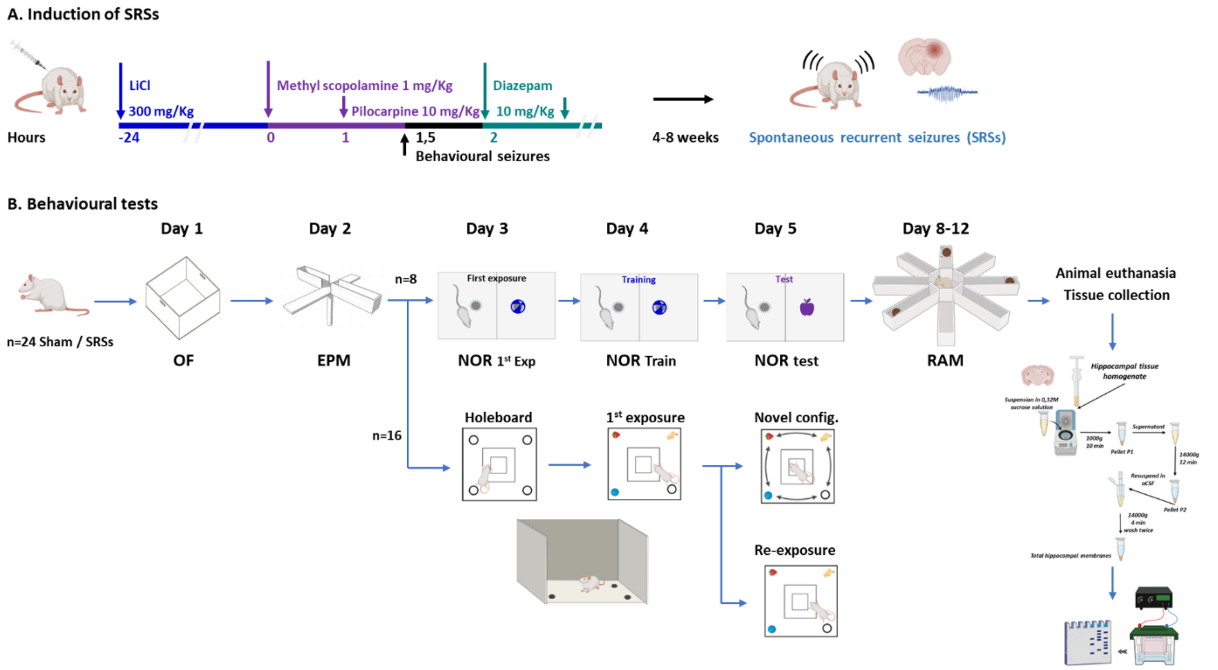

Supplementary Figure S1 – Experimental workflow for induction of SRSs with Li^{2+}-pilocarpine in the rat (A.) and for behavioural evaluation (B.). In **A.**, the sequential drug treatments required to induce and terminate behavioural seizures are shown in the left and the development of spontaneous recurrent seizures (SRSs) within 4-8 weeks are shown on the right. In **B.**, the sequence and division of animals to the different tests is shown sequentially. EPM – elevated plus maze test; NOR – Novel Object recognition test; OF- Open field test; RAM – radial arm maze test for spatial memory.

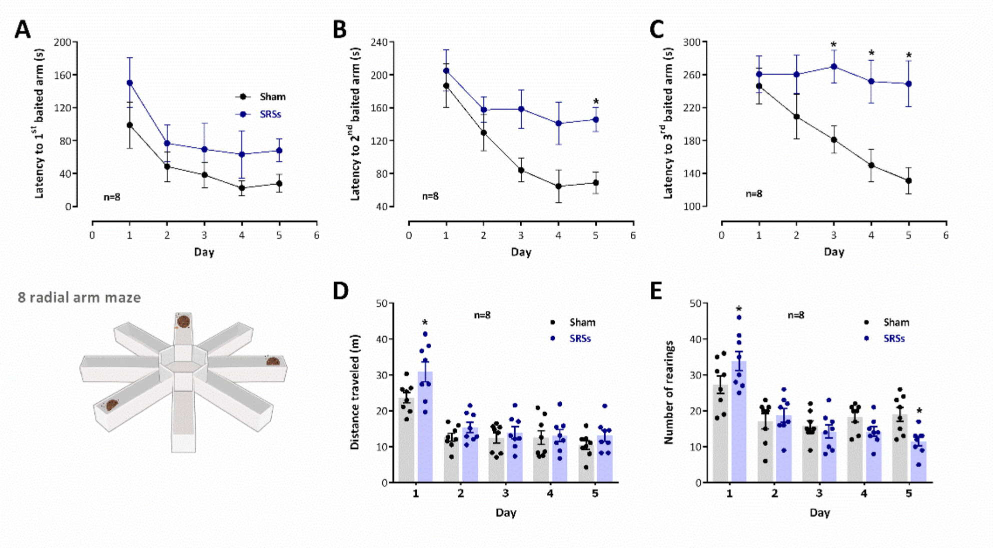

Supplementary Figure S2 – Impaired learning in the radial arm maze in the Li^{2+}-pilocarpine rat model of epilepsy. Learning performance in the RAM was evaluated by the latencies to find the first (**A.**), second (**B.**) and third (**C.**), baited arms. Global exploratory activity in the RAM was evaluated by the total distance travelled (**D.**) and number of rearings (**E.**) during each trial. Total trial duration was of 5 min for each session. A schematic representation of the RAM apparatus is depicted (bottom, left) showing example of one possible configuration for the three baited arms. Values are the mean ± S.E.M. *$p < 0.05$ vs Sham controls (Two-way ANOVA).
